# Effect of Upright and Slouched Sitting Postures on the Respiratory Muscle Strength in Healthy Young Males

**DOI:** 10.1155/2018/3058970

**Published:** 2018-02-25

**Authors:** Ali Albarrati, Hamayun Zafar, Ahmad H. Alghadir, Shahnwaz Anwer

**Affiliations:** ^1^Department of Rehabilitation Sciences, College of Applied Medical Sciences, King Saud University, Riyadh, Saudi Arabia; ^2^Rehabilitation Research Chair, College of Applied Medical Sciences, King Saud University, Riyadh, Saudi Arabia

## Abstract

**Objective:**

The present study compared the effects of upright and slouched sitting postures on the respiratory muscle strength in healthy young males.

**Methods:**

A total of 35 adult male subjects aged 18–35 years participated in this study. Respiratory muscle strength was determined by measurement of sniff nasal inspiratory pressure (SNIP) using a MicroRPM device in the upright and slouched sitting positions. The subjects were asked to perform the pulmonary function test including peak expiratory flow (PEF), forced expiratory volume in one second (FEV_1_), forced vital capacity (FVC), and FEV_1_/FVC ratio at baseline. Body composition was also determined.

**Results:**

There was a significant difference of SNIP score between upright sitting and slouched sitting positions (*p* = 0.04). The mean difference of SNIP score between upright sitting and slouched sitting positions was 8.7 cmH_2_O. Significant correlations were found between SNIP in upright sitting and FEV_1_% predicted values [*R* = .651], SNIP in slouched sitting and FEV_1_% predicted values [*R* = .579], and SNIP in upright sitting and SNIP in slouched sitting positions [*R* = .926] (*p* < 0.05 for all). There were no significant correlations between SNIP scores, demographic variables, and other baseline clinical data (*p* > 0.05).

**Conclusions:**

The slouched sitting position had a lower SNIP score compared to upright sitting position suggesting a reduced diaphragm tension and movement as a result of altered body posture.

## 1. Introduction

Altered body position influences the respiratory muscle strength and function in both healthy adults [1–5] and patients with cardiopulmonary dysfunction [6, 7]. A study by Costa et al. [1] reported significantly lower maximal inspiratory and expiratory mouth pressures in supine or semiupright sitting positions compared to the sitting position in healthy young adults. Similarly, Koulouris et al. [2] reported reduced respiratory muscle strength in the supine position compared to sitting position. Biomechanically, the length of the muscle affects the ability of a muscle fiber to develop active tension known as length-tension relationship [8]. Therefore, it is assumed that the changes in the ribcage may cause altered length-tension relationship of the respiratory muscles, such as diaphragm, resulting in reduced ability of these muscles to develop tension and consequently reducing the rate and depth of the breathing [1].

The measurement of the respiratory muscle strength is vital in the evaluation of therapeutic effects of various interventions for the respiratory muscle weakness or dysfunction [9]. Inspiratory muscle strength can be measured using a simple, reliable, and valid test known as sniff nasal inspiratory pressure (SNIP) [10–13]. The SNIP is a noninvasive, easy, and more acceptable technique compared to the static effort of the maximum inspiratory pressure [13] and has been an alternative [12, 14] to the measurement of the maximal inspiratory pressure.

The body position has a vital role in the cardiopulmonary physical therapy. Several positions, including sitting, supine, side lying, and semi-Fowler positions, have been adopted by the patients during the treatment sessions [1]. Nevertheless, these positions could influence the performance of the respiratory muscles during therapeutic interventions. This is especially applicable as the improvement of the inspiratory muscles' strength in respiratory conditions, which are likely to become weak, is an essential outcome measure for physiotherapists [1]. The present study aimed to compare the effects of upright and slouched sitting postures on the respiratory muscle strength in healthy young males.

## 2. Methods 

### 2.1. Subjects

A convenience sampling technique was used to recruit subjects from the College of Applied Medical Sciences. Subjects were apparently healthy and free from cardiopulmonary disease and the inclusion criteria were as follows: adults aged between 18 and 35 years as the most obstructive pulmonary disease occurs in adults above the age of 35 years [15, 16], no restriction on the type of physical activities, body mass index (BMI) ≤ 30, and adults free from any lung diseases as documented by the spirometry. Subjects were excluded if they had a history of surgery at thoracic vertebra, recent pulmonary embolism and deep vein thrombosis, chest disease, low back pain, and a spinal fracture and congenital spinal deformity, for example, kyphosis. The study was approved by the Institution of Ethics Committee of Rehabilitation Research Chair, King Saud University. Each subject signed a written informed consent form approved by the Institution Ethics Committee, before participation. All experiments followed the Declaration of Helsinki.

### 2.2. Anthropometry Measurement

Height and weight were measured with subjects wearing lightweight clothing and barefoot using a stadiometer and body mass index was calculated in kg/m^2^.

### 2.3. Lung Function Tests

All subjects performed pulmonary function test including peak expiratory flow (PEF), forced expiratory volume in one second (FEV_1_), forced vital capacity (FVC), and FEV_1_/FVC ratio using portable Vitalograph device (Vitalograph, Ltd., UK) according to the guidelines of the American Thoracic Society [17].

### 2.4. Inspiratory Muscle Strength Measurement

**Figure 1:**
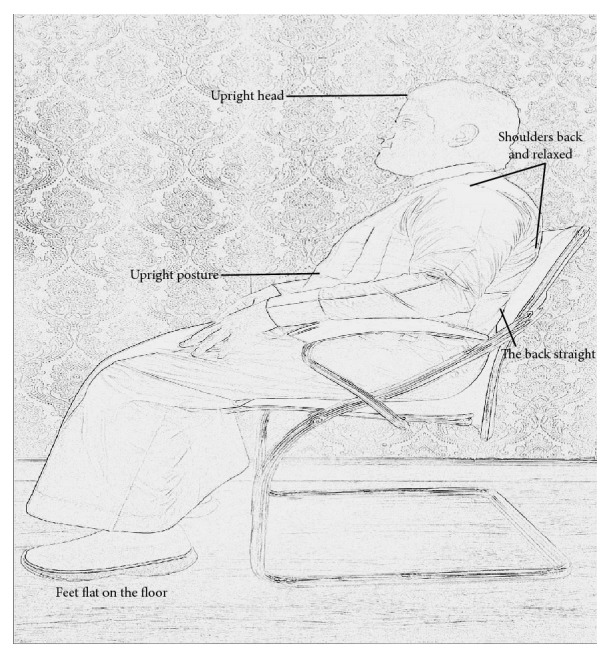
Upright posture.

**Figure 2:**
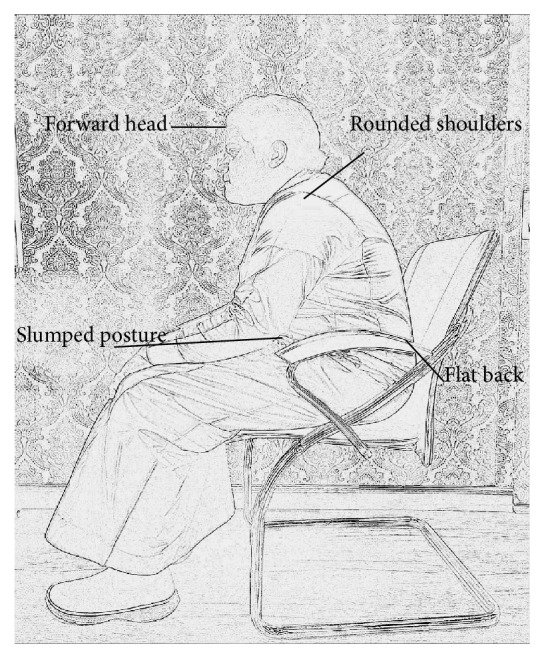
Slouched posture.

Sniff nasal inspiratory pressure (SNIP) was measured as per the previously described methods [12] using a MicroRPM device (MircoRPM, MicroMedical, Ltd., Kent, UK). Subjects were asked to sit in upright posture with upright head, shoulders back relaxed, and feet flat on the floor (Figure 1). Then, subjects were asked to sit with forward head, rounded shoulders, slumped posture, and flat back with feet rested on the floor (Figure 2). Standardized verbal commands were given to maintain each posture. In the first position (upright sitting), the subject took a sharp and quick sniff from functional residual capacity (FRC). This maneuver was repeated for at least five times or until the subject could not score more than 10 cmH_2_O and the maximum value was chosen [18]. The subjects repeated the same procedure in a slouched sitting position. Both the sitting positions were randomized to avoid order effect.

### 2.5. Statistical Analysis

The Statistical Package for Social Sciences (SPSS) for Windows (Version 22.0, SPSS, Inc., Chicago, IL) statistical software was used for all statistical analyses. Normality of the data was determined prior to the analysis. Descriptive statistics (mean and standard deviation) were used to summarize the data. Paired* t*-test analysis was calculated to identify differences of SNIP scores between upright sitting and slouched sitting positions. Pearson correlation test was used to investigate the relationships between SNIP score on two positions and the demographic variables and the baseline clinical data. The significance level (*p* value) was set at 0.05.

## 3. Results

A total of 35 subjects participated in this study.  Table 1 details the demographic variables and baseline clinical data.  Table 2 presented the comparison of SNIP score on two different sitting positions. The subjects had lower SNIP score during a slouched position compared to normal upright position (*p* = 0.04). The mean difference of SNIP score between the upright sitting and slouched sitting position was 8.7 cmH_2_O. Significant correlations were found between SNIP in upright sitting and FEV_1_% predicted values [*R* = .651], SNIP in slouched sitting and FEV_1_% predicted values [*R* = .579], and SNIP in upright sitting and SNIP in slouched sitting positions [*R* = .926] (*p* < 0.05 for all). There were no significant correlations between the SNIP scores, and age or BMI or other baseline clinical data (*p* > 0.05).

## 4. Discussion

The present study aimed to compare the effects of body postures during upright and slouched sitting positions on the inspiratory muscle strength in the healthy young males. The results of the present study demonstrated that altered posture during slouched position reduced the diaphragm strength as measured by SNIP compared to normal upright position. In the present study, a drop of 9.3% in the SNIP measurement during the slouched sitting position compared to the upright sitting was reported.

Biomechanical alteration of postural alignment affects the ranges of motion, position, and coupling patterns of the articulations between the thoracic spinal vertebrae and ribcage, which influence lung compliance via changing articular movement available for breathing [19]. The diaphragm has several attachments to spinal vertebrae and ribcage and changes in the position of these bony structures altered the proper function of the diaphragm. Like other skeletal muscles in the body, the diaphragm contracts and relaxes in order to maintain proper breathing mechanics and also contributes significantly to spine stability and ribcage movement. Restriction of the ribcage during slouched position limits the mobility of the diaphragm which subsequently and unconsciously induces breathing disorder [20, 21]. In addition, slouched position contributes to impairment of other systems including reduced venous return, autonomic nervous system, and phrenic nerve excitability. Similar to our study, previous studies have reported an increased respiratory effort and reduced respiratory capacity and control in normal individuals in a slouched position compared to normal erect sitting position [20, 22, 23].

Facilitating a normal breathing pattern needs an effective diaphragm muscle contraction [21]. Adapting a slouched position reduces the ability of the diaphragm to generate appropriate force for contraction. This attributes to restriction imposed by the abdominal cavity. This is supported by a number of studies which demonstrated an alteration of the ribcage and the diaphragm strength force during different positions [1, 21, 24]. A study by Lee et al. [21] using respiratory inductive plethysmography (RIP) had demonstrated variations in the thoracic and abdominal cavity characteristics in different habitual sitting positions. Furthermore, Kera and Maruyama [24] and Lee et al. [21] reported a decreased muscle activity in a slouched sitting position compared to more upright sitting position. Moreover, using similar methods to our study, Costa et al. [1] reported significantly lower maximal inspiratory and expiratory mouth pressures in supine or semiupright sitting positions compared to the sitting position in healthy young adults.

In the present study, the higher SNIP score in upright sitting position compared to slouched sitting position may be due the fact that in more upright position the diaphragm had a mechanical advantage and more favorable positions in the length-tension curve to create tension [3]. In addition, the length-tension relationship of all other inspiratory muscles may become altered in slouched sitting position to produce optimal muscle tension.

The present study demonstrated a little higher positive correlation between the SNIP score in upright sitting position and FEV_1_ predicted values compared to the SNIP score in slouched sitting position. This is supported by a previous study that suggested better spirometry outcomes in the upright position than supine position in healthy individuals [25]. However, in the present study, other spirometry outcomes including PEF, FVC, and FEV_1_/FVC ratios showed insignificant correlation with the SNIP scores in either position.

The present study demonstrated insignificant correlations among SNIP scores and the demographic variables such as age, height, weight, and BMI. However, a previous study reported that the demographic factors such as age, weight, BMI, and height influence the inspiratory muscle force in healthy individuals [26]. Another study reported a negative correlation between age and SNIP score in men and a positive relation between BMI and SNIP scores in women [27]. Several factors contributed to these differences. First, the possible reason is the different posture. Second, in the current study, subjects were young where the effect of age on the diaphragm is unlikely. Third, the lack of correlations might be attributed to small sample size in the current study.

The present study had some potential limitations. The result of the present study was limited to healthy young males. The comparison of the lung function in different postures was not measured to document the effect of slouched position on lung volumes. In addition, the lack of comparative group limits the validity of the present study. Furthermore, quality trials investigating the effect of changing posture on respiratory muscle strength in patients with breathing disorders are recommended.

## 5. Conclusion

The slouched sitting position had a lower SNIP score compared to upright sitting position suggesting a reduced diaphragm tension and movement as a result of altered body posture. Prolonged slouched position may induce breathing disorder and affect surrounding structures including the heart and phrenic nerve. Individuals are advised to avoid slouched position and encouraged to practice upright position with proper breathing maneuvers. Future studies should look at the effect of reversing chronic slouched position on the diaphragm and lung volumes.

## Figures and Tables

**Table 1:** Participant's characteristics and baseline data.

Variables	Mean (SD)
Age (years)	22.5 (5.6)
Height (m)	1.7 (.05)
Weight (kg)	74.6 (16.04)
Body mass index (kg/m^2^)	25.6 (4.04)
SNIP_Sitting_ (cmH_2_O)	93.5 (25.8)
SNIP_Slouched_ (cmH_2_O)	84.8 (22.5)
PEF (%)	.90 (.13)
FVC% predicted	.85 (.09)
FEV1% predicted	.98 (.13)
FEV1/FVC ratio	115.7 (17.7)

PEF: peak expiratory flow; FVC: forced vital capacity; FEV1: forced expiratory volume in one second; SNIP_Sitting_: sniff nasal inspiratory pressure in upright sitting; SNIP_Slouched_: sniff nasal inspiratory pressure in slouched sitting.

**Table 2:** Sniff nasal inspiratory pressure in different body positions.

	SNIP_Sitting_ (cmH_2_O)	SNIP_Slouched_ (cmH_2_O)
Mean	93.5	84.8
Standard deviation	25.8	22.5
Mean difference	8.7	
SD_diff_	9.8	
95% confidence interval of the difference	3.2–14.1	
*t*-value	1.414	
*p* value	0.04^*∗*^	

SNIP_Sitting_: sniff nasal inspiratory pressure in upright sitting; SNIP_Slouched_: sniff nasal inspiratory pressure in slouched sitting; ^*∗*^*p* < 0.05 was considered statistically significant.

## Abbreviations

SNIP: Sniff nasal inspiratory pressure
PEF: Peak expiratory flow
FEV_1_: Forced expiratory volume in one second
FVC: Forced vital capacity.
